# 122nd Annual Meeting Medical Library Association, Inc. New Orleans, LA May 3–6, 2022

**DOI:** 10.5195/jmla.2023.1707

**Published:** 2023-04-21

**Authors:** JJ Pionke, Ellen M. Aaronson

**Affiliations:** 1 pionke@illinois.edu, Proceedings Co-editor, Applied Health Sciences Librarian, University Library, University of Illinois at Urbana-Champaign, Urbana, IL.; 2 aaronson.ellen@mayo.edu, Proceedings Co-editor, Librarian, Mayo Clinic Libraries, Rochester, MN.

## INTRODUCTION

The Medical Library Association (MLA) held its 122nd annual meeting May 3-6, 2022, in New Orleans, Louisiana. The meeting was entitled “MLA '22: Reconnect. Renew. Reflect” and utilized a hybrid model with some events in person, and some virtually. The virtual meeting was again broken into segments, all available using a variety of online platforms. Total attendance for the meeting was 1,250 with 575 attending in-person, and 675 virtually.

Additional meeting content—including the meeting program and various electronic presentations from the business meetings, plenary sessions, poster sessions, and program sessions can be accessed by all meeting registrants via the MLA '22 website.

## OPENING SESSION (VIRTUAL)

Wednesday, April 27, 2022, 10:30 a.m. - 11:45 a.m., central time

Kristine M. Alpi, AHIP, FMLA, 2021/2022 MLA President

Emily Emily J. Hurst, MLA ‘22 Cochair

Lisa A. Marks, AHIP, MLA ‘22 Cochair

Kris Alpi: Good morning. Thank you all for being with us today. I am Kris Alpi, your MLA President, and host for this opening session. It's great to share this virtual stage for the kick-off session of the MLA 122nd Annual Conference and Exhibition. I am very excited to see you online now and soon, many of you in-person in New Orleans since this is a hybrid meeting! The first 119 MLA conferences were held in person, and as many of you know, our 2020 and 2021 conferences were both held virtually because of the Covid-19 pandemic. That was quite a change, and we all met the challenge to develop and participate in a different kind of meeting experience.

Before we talk about this year's Annual Conference, I want to emphasize that thanks to quality programming and our innovation in the virtual experience of attendees and exhibitors, the 2020 and 2021 conferences involved more of you than our typical in-person conference attendance of 1,050 or so. More than 1,100 of you attended the 2020 conference, and more than 1,200 of you attended in 2021. This year, with Covid-19 infection rates lower, and higher vaccination rates, the Omnipress 2022 State of the Conference Industry Report indicates that 88% of associations are offering an in-person component to their meeting, 1% a virtual-only meeting, and 11% have not yet decided. MLA is one of the 29% of associations holding an event with both a virtual and in-person component. The main drivers of our robust virtual component are accessibility and inclusion, especially considering the tight budgets you and your organizations may be facing.

As we approach the May 3 opening of our in-person component, we all continue to experiment and navigate as best we can and stay alert for what may come next. We keep learning, we adjust, and we move forward, always with public health and care for each other in mind.

Many of you have shared with us how much you miss connecting in-person at meetings where you get together with colleagues, participate in onsite immersion and poster sessions, and take in the sights, sounds, and smells of the host city. What better place to bring us back together than New Orleans - a city bursting with revival, energy, great music, history, fabulous sightseeing opportunities, and exquisite Creole cuisine. We look forward to welcoming you all next week.

For those of you who are unable to attend in-person, the National Program Committee and Headquarters' staff have planned yet another new meeting format with a robust and interactive virtual component.

I am happy to report that total attendance in 2022 will meet or exceed prior year levels:

- More than 1,063 of you have already registered, with 550 attending in-person. We expect total registration to be as high as 1,250.
- 500 of you are attending thanks to the outstanding commitment of 60+ institutions that purchased group registrations.
- More than 170 exhibitor representatives will be onsite for the exhibit floor and vendor sessions.

Yes, if you are attending today to learn more about the meeting, you can still register so that you do not miss out on keynotes, contributed content, exhibits and vendor programming, and everything else the 2022 National Program Committee (aka the NPC) has planned. If financial hardship is holding you back, we continue to offer no cost registration for the virtual meeting at no cost. You will find the application form in the Financial Support link of the meeting registration menu.

### Agenda

Kris Alpi: Let's go over what is coming up in the rest of today's event:

1. MLA Statement of Appropriate Conduct - This MLA experience should be open, inclusive, and accessible to all. We will review our rules and guidelines as the first agenda item.
2. Reflections - We will reflect on issues that have touched us all and that continue to occupy our minds as we gather today. I will also announce this year's recipient of the MLA Award for Distinguished Public Service.
3. Recognition of Sponsors - We will hear recorded messages from our Platinum and Gold partners throughout this session.
4. MLA'22 experience - Emily J. Hurst and Lisa A. Marks, co-chairs of the 2022 National Program Committee, will recognize those who made this meeting possible. They will also give us tips on how to best experience the next few days.
5. Presidential address - I will briefly share perspectives and offer gratitude on my year as MLA President.

This year, we will again have a virtual Awards and Recognition Celebration. Rather than including it in this opening session, we will dedicate a special event that will take place virtually on June 14, 2022. This is part of our effort to distribute important MLA activities throughout the year rather than try to fit them all into the hectic weeks around the annual conference.

In this June 14 session, we will:

- recognize and celebrate your many accomplishments and contributions to the association and profession, and
- remember our colleagues who have passed away over the past year.

We look forward to enjoying the inspiring videos and photos that our 2022 awardees are preparing or have already provided. Please mark your calendars, and tell your co-workers, family, and friends as this session will be open to all.

### MLA Statement of Appropriate Conduct

Kris Alpi: The MLA Board of Directors approved the MLA Statement of Appropriate Conduct in 2020. It applies to all MLA activities including conferences, meetings, workshops, online forums, social media, continuing education, and all means of communication relating to MLA activities. It applies to all MLA members, non-members, invited guests, speakers, moderators, instructors, exhibitors, staff, and all others who participate in an MLA activity or event.

Our objectives are:

- an open, inclusive, and collaborative environment within the association in all its activities and events, and outside the profession.
- diversity, equity, and inclusion in professional practice, leadership of health sciences libraries, and information professionals.
- advancement and support of accessibility for all stakeholder groups; and
- irreproachable ethical standards that call for health sciences librarians to conduct all professional relationships with courtesy and respect.

Please read the statement which is located on the MLA website under About MLA. It includes the do's and don'ts, how to report inappropriate behavior, and the resolution process.

At the bottom of the statement, there is a link to an incident reporting form.

### Reflections

Kris Alpi: Let's now reflect on issues that have touched us all and that continue to occupy our minds as we gather today: The Covid pandemic has had a devastating effect on physical health, mental health, financial wellbeing, exclusion, disparities, and more. With over 6 million deaths as of April 6, 2022, including close to 1 million in the US alone, we recognize we all have been impacted either personally, with family and friends, or through our work (https//coronavirus.jhu.edu).

In addition to the losses resulting from the COVID-19 Pandemic, the war in Ukraine has created destruction and pain for the citizens of Ukraine and raised the level of tension and fear worldwide. Earlier this year, MLA issued a statement in support of the Ukrainian Library Association and the people of Ukraine and condemning Russia's aggression and violence and we hope for a resolution of the conflict that is tearing lives apart. Through our International Cooperation Caucus and Health Equity & Global Health Domain Hub and through relief efforts at many institutions, MLA members have moved beyond the statement to offer concrete support to our colleagues in Ukraine.

We have an individual and group responsibility to end systemic racism and fully embrace diversity, equity, inclusion and belonging. We strive to create a just, civil, and sustainable society for today and for future generations. The statement calling for greater access to gender-affirming healthcare for all transgender individuals and especially transgender youth. advanced by the LGBTQIA+ Caucus, frames that commitment and puts out a call to action.

Thank you to our many caucuses and chapters who have signed onto the Ukraine and LGBTQIA+ Caucus statements, and to the individual members who are active participants in this journey.

MLA continues to develop its voice as health information professionals and respond to the sustained attacks on evidence-based medicine and research in the United States which undermine our institutions, particularly those essential to the quality of healthcare. We also stand with the American Library Association in the fight against censure of books, and with every library staff member on the front lines of this challenge to share information for everyone.

Thank you for engaging in dialog and advocacy that strengthens our communities and makes us better. Now I want to introduce an outstanding public servant who has tremendously impacted sharing information and advancing knowledge.

### Distinguished Public Service Award

Kris Alpi: Earlier this year, the Board of Directors bestowed the MLA Award for Distinguished Public Service on Dr. Francis Collins, Director of the National Institutes of Health from 2009 - 2021.

This MLA award was established in 1988 to honor persons whose exemplary actions have served to advance the health, welfare, and intellectual freedom of the public.

Dr. Collins has a strong connection with MLA and issues and policies important to the health sciences library community. He presented the 2002 NLM/MLA Joseph Leiter Lectureship speaking on Genomics, Medicine, and Society. Dr. Collins launched the All of Us Research Program which has enrolled almost 1 million people across the US to share their health data so researchers can improve how we prevent illness and treat diseases and conditions, and the Helping to End Addiction Long-term (HEAL) to address the opioid crisis by improving treatments for opioid misuse, addiction and pain management.

Most recently Dr. Collins engaged MLA and other associations in the NIH listening sessions on the Advanced Research Project Agency for Health (ARPA-H).

In presenting this award to Dr. Collins, MLA especially recognizes his passion to ensure public access to the knowledge that advances science and impacts health.

In her video tribute, Patti Brennan described Dr. Collins as a leader with “passion, vision, determination, and principle.” As the longest serving presidentially appointed NIH Director, Dr. Collins has steered the country's largest medical research agency with a calm hand, a scientific mind, and a deep commitment to the well-being of all Americans.

These two photos illustrate Dr. Collins' ties with the Medical Library Association.

On the left is a photo of 2009-2010 MLA President Connie Schardt, AHIP, FMLA engaging in conversation with Dr. Collins at his welcome reception following his installation as NIH Director.

The photo on the right captures Dr. Collins with Kate Flewelling, AHIP and Veronica Milliner in front of Abyssinian Baptist Church, Harlem, at the official launch of the All of Us Research Program in 2018. Flewelling and Milliner represented what is now known as the Network of the National Library of Medicine at the event.

We will share more about Dr. Collins when we host MLA's 2022 Awards and Recognitions Ceremony on June 14, 2022. Please plan to join us at that celebration which is virtual and open to all.

Creating an open, inclusive, and collaborative environment starts with each of us. MLA exists because we want to connect with each other, grow our knowledge and skills, and amplify voices in our profession to achieve change. Visit “I am MLA” on the MLA website home page to learn our stories and share your own as an “I am MLA ambassador.” The site includes 55 stories, with new ones added regularly.

### Exhibitor and Sponsor Recognition

Kris Alpi: Please join me in giving a heartfelt welcome and thank you to our sponsors and exhibitors who once again demonstrated their outstanding support for MLA, and the value they see in health information professionals.

They contribute to MLA in so many ways and help us thrive

- In 2021, exhibitors, sponsors, and advertisers provided $477,000 or 21% of MLA operating revenues.
- Exhibitors, sponsors, and advertisers are back in full swing for this 2022 conference, and we are so grateful. They are looking forward to seeing us. In addition to the exhibit hall and sessions, there will also be vendor activities and connections for our virtual attendees, stay tuned for more on that later in this session.

Vendors also engage and collaborate with MLA members on initiatives that advance our knowledge sharing and our profession.

The MLA InSight Initiative was a great example of that collaboration. InSight was a thought-leadership initiative, engaging health sciences librarians, publishers and vendors in high-level, high-value dialogue on issues that matter to our communities. The premise of InSight was to build goodwill and collaboration between the health sciences librarian and health information provider communities. Since its 2018 launch, InSight participants collaborated and organized six summits and a Fall Forum, with individuals from both the librarian and the publishing communities planning the meetings and the follow-up workgroups. InSight reinforced, expanded, and communicated the value that our communities contribute in moving validated information from the author to the reader. In 2021, the InSight steering committee recommended to sunset InSight as a separate program and integrate the energy of its participants and its successful outcomes into expansions of other MLA activities and communities. InSight laid the groundwork for the Development of MLA's first Collection Development and Resource Sharing Symposium being held May 4-6 in New Orleans. The symposium sessions highlight MLA's commitment to health information professionals at all levels of experience and knowledge who serve in the areas of collection Development and resource sharing. This program was designed as an interactive, in-person experience leveraging the engagement of publishers attending the meeting. After the symposium, we will strategize on what's next based on what we learn in New Orleans.

Kris Alpi: Let's take time to thank our annual conference sponsors who have contributed support ranging from $5,000 to more than $45,000. We are grateful for their support and invite you to take time to personally thank them for their support throughout the meeting and afterwards.

Our Bronze-level sponsors, in alphabetical order, are:

- Cabells [CABells - pronounce CAB like you would a taxicab]
- Covidence
- Elsevier
- Elsevier Health
- Rittenhouse
- Slack Journals
- Springer Nature
- Wiley

Our Silver-level sponsors are:

- Clarivate
- JAMA Network

Our Gold-level sponsor is:

- New England Journal of Medicine

Let's Hear from Eric J. Rubin, M.D., Ph.D. Editor-In-Chief New England Journal of Medicine

[Recorded message from Dr. Rubin]

Thank you, Dr.Rubin, and thank you to the very supportive New England Journal of Medicine team.

We are especially grateful to our Platinum-level sponsor, Wolters Kluwer

Let's hear from Vikram Savkar [VICK-ram SAV-car] Vice President & General Manager, Medicine Segment Health Learning, Research & Practice on behalf of Wolters Kluwer

[Recorded message from Savkar]

Vikram Savkar: On behalf of all of us at the Medical Library Association, we thank you for your support and that of the Wolters Kluwer team.

### MLA'22 Experience

Kris Alpi: Are you ready to talk about this year's conference? We are! We are energized by the enthusiasm of our members, organizers, presenters, MLA staff, and technology partners who have been so committed in preparing this meeting. We salute the hard work and vision of the organizers for their creativity, building on the success of last year's virtual conference to design a “new and improved” experience for all of us to enjoy. The word “awesome” comes to mind for both the amount of effort involved and the fabulousness of the content that is coming your way. Here to talk to you about the awesome virtual conference you are about to experience, and the extra-awesome in-person conference in New Orleans, please welcome your NPC22 co-chairs, Emily Hurst and Lisa Marks, AHIP.

Emily Hurst: Thank you Kris. I am Emily Hurst, your NPC22 Co-chair.

Lisa Marks: And I am Lisa Marks, your NPC22 Co-chair.

Emily Hurst: On behalf of the entire National Program Committee, welcome to “MLA ‘22: Reconnect, Renew, Reflect.”

### Conference Theme and Objectives

Emily Hurst: The theme says it all, and we are so happy to be bringing back the in-person conference, after two years of Zoom. We know you can't all make it to New Orleans, and we will miss you. That's why this year, we went even further in our conference design: a full virtual experience AND a full in-person experience. Some call it “hybrid” - we call it… a lot more work, but totally worth it.

Lisa Marks: Emily - when we signed up as co-chairs, I had no idea that we would be redesigning a conference for the third year in a row. Kudos to the amazing MLA headquarters team and the conference team for helping us pull this off. I have just received the count for this session: 195 are watching us live so far, with a steady flow coming online as this gathering continues!

### NPC Recognition

Emily Hurst: Our NPC ‘22 team has been extraordinary. Please join Lisa and me in recognizing them. (Photos first, and then names.)

Lisa Marks: We also want to recognize the MLA members and representatives of our publishing partners who assembled an impressive pilot program on Collection Development and Resource Sharing as it relates to Health Sciences. We believe that MLA can provide new value to those of us in that area of specialty, and we look forward to this quality program in New Orleans next week.

Emily Hurst: When you work so closely with the MLA Headquarters team to organize this conference, you get a real appreciation for the breadth and complexity of the task. The MLA Headquarters team is running two meetings at once, plus a symposium, while ensuring that your experience in each is as best as can be. They also support the exhibitor presence and programming. Please join us in showing our appreciation for what they do. Thank you.

### Conference Overview

Emily Hurst: Here's what's in store for this year's conference. Registered attendees have already been exploring the online planner. Before the end of this session, most of the on-demand content will be available for you to view. Lisa and I will provide a few tips about the planner and how to best experience MLA ‘22. A reminder that you will need to upload a photo in your profile; if you do not choose to use your own, your pet, a favorite plant, a shiny object–or pictures offered by MLA of the sun and planets are options for you (see the chat for the URL). Now, let's talk about some changes for this meeting (yes, more change!) For the first time, we are introducing virtual paper and lightning talk sessions, since we know not all presenters could attend MLA ‘22 in person. On your menu, select the down arrow to open presentations in a session; for virtual sessions, the “join session” button will be the same for all presentations in a session. And while most presenters have already posted content online, we also know that you cannot always commit to watch presentations ahead of time. We also so appreciate the presenters who added their videos or slides for you to view on-demand–since concurrent sessions are back–and you WILL need to make decisions about what to attend live. And please don't miss NLM/NNLM Day on April 28!

Lisa Marks: Emily, I have already found I have to make decisions about what to attend! But one thing introduced last year and continuing especially for our virtual attendees and sessions, is… time zone support! When you first log into the Online Planner, make sure to put in your time zone. All live sessions then show in YOUR time zone so you can plan your time effectively. We've also added some detailed instructions for how to add presentations to your Outlook calendar, so you can get the reminders you need while you are still at work. Anything you favorite (select the star by the presentation) can be added to your calendar and you can add personal meetings too. If you are attending the New Orleans meeting, you may wish to revert your time zone to Central time while you are onsite and use the app to keep track of your schedule.

Emily Hurst: Lisa, thanks for mentioning the App! The app is available this year, and if the download link and QR code is not up on the site now, it will be shortly. The app will let you play MLA ‘22 Quest and keep track of everything onsite you want to see. Two last changes I want to point out: the My Experience page in the online planner is your one-stop-shop for all your favorites, questions, attendee network, MLA ‘22 Quest, system checks, favorite exhibitors, and more. And in an MLA first, we will be live streaming all three main plenary sessions out to virtual attendees. We do apologize that for our West Coast, Alaska, and Hawaii attendees, the early hour for these plenaries may mean you wish to instead watch the post-session recordings.

Lisa Marks: Emily, these are all game-changers for a hybrid conference–I can see I need to carefully go through this schedule!

Emily Hurst: Lisa, you're right–but now why don't you tell us how our attendees in New Orleans will be experiencing all the great MLA ‘22 content onsite.

Lisa Marks: Certainly!

- On Tuesday, our onsite meeting begins with registration in the morning and the LAC's hospitality center and continuing education courses. When you register onsite, please be sure to have your vaccination card (or a picture of it) to show at the Registration desk.
- On Wednesday, we kick off with an in-person New Member/First Time attendee event (and a grab and go attendee breakfast pickup, followed by our welcome and the Janet Doe lecture with our colleague Michael Kronenfeld. The Collection Development & Resource Sharing Symposium begins with a workshop on negotiating strategies that are applicable across libraries and life. We'll start the afternoon with a lunch for all attendees, celebrating our communities and hearing more from Kris Alpi and Adela Justice about the community transition that has happened over the pandemic and this year's community assessment. Then we're off for an afternoon of programs until the Welcome Reception and Opening of the live Hall of Exhibits.
- After either a night of reconnecting, or a good rest, on Thursday we'll have exhibitor Sunrise Seminars ahead of the John P. McGovern Lecture with Dr. Mona Hanna-Attisha. Dr. Mona will also have a book signing before exhibitor lunch and learn sessions, an in-person poster session and an afternoon of programs, technology showcases, a Vision 2048 working session, and an in-person Living Library event.
- Friday, we begin the day with an in-person NLM Update, followed by more programming, a second poster session, more exhibitor events, and your last chance to visit the Hall of Exhibits before a snack break, our short Closing session, and an afternoon to explore a bit more of New Orleans.

Emily Hurst: Lisa, that is a very busy set of days!

Lisa Mark: And Emily, I know that you, as an incoming board member of MLA, will definitely be spending time thanking our sponsors and exhibitors in the Hall.

Emily Hurst: [Virtual nodding and thumbs up]

Lisa Marks: MLA ‘22 has a LOT of programming, and I can't wait to get started this afternoon. We really thank our speakers and contributed content presenters for being so flexible and for going the extra mile to make MLA ‘22 a success for both our virtual and in-person attendees. Your participation is essential to strengthening our community.

Emily Hurst: This concludes the part of this session on how to experience MLA'22. We look forward to your feedback. It is now my great honor and pleasure to transition to the next and final segment of today's program: the Presidential Address. Kris Alpi has invested her energy in being an inspiring and caring leader in her tenure as MLA President in yet another challenging year. Please join me in congratulating Kristine Alpi, AHIP, FMLA and MLA's 2021-22 President and welcoming her to present her Presidential Address.

## OUTGOING PRESIDENTIAL ADDRESS

Kris Alpi: Hello again, everyone. Thank you for staying for the last segment of today's session to hear how MLA has advanced during my presidential year.

Here's the playbook for the next 20 minutes. I'd like to introduce some of the many teams that I've worked with this year and celebrate and recognize the efforts of several individuals that shaped my presidency.

As we kick off MLA's virtual meeting, I would like to highlight a few amazing virtual conferences of health sciences information professionals where I was invigorated by our regional and global relationships.

I have a bit more to say about some wonderful programs that have advanced to broader and more sustainable approaches. Then I will wrap up with work in progress that I hope to advance as Past-President under Shannon Jones' leadership. First and foremost, I thank my colleagues at Oregon Health & Science University. Many of you probably know some of the people pictured here, but I am going to highlight four people who are not MLA members. For anyone who has met with me this year and had to play calendar games, you know how important Maia Siufanua pictured at the top in the yellow cardigan has been. In addition to her full-time position supporting everyone else in the Library, she has worked with team MLA headquarters to perform scheduling magic. My supportive boss, interim provost. Dr David Robinson is shown to the far right. Without his generosity, I would not have had the time for all of the activities we will discuss today. Below his picture, just to the left, meet Tracy Thornton, Director of Library Operations, who keeps everything running in the library when I was living across so many time zones this year. And finally, thank you to my colleague, Shannon, at the bottom, who put this lovely collage of our team together.

Thank you, team OHSU Library!

I'm so excited about this next part. Each year, the MLA President has the honor of recognizing colleagues who have enhanced the health sciences library profession and furthered the objectives of the association. Recipients of the President's Award receive a unique MLA lapel pin. When we are together in-person or on video in the future, I hope they will wear their pins proudly.

Now, it is my privilege to present the MLA President's Award for 2022. I'm excited to recognize the Hospital Library Advocacy Team with MLA's 2022 President's Award. This team of ten Hospital Library Caucus members collaborated with MLA Headquarters staff to develop and launch MLA's hospital library promotion and advocacy strategy. On behalf of the Board and MLA members, we thank the following members for creating a website that features resources for hospital and health care administrators highlighting the mission-critical roles that health sciences information professionals provide in healthcare settings. for developing the statement, “Partner with Hospital Libraries to Improve Patient Care,” and for updating MLA's Hospital Library Standards.”

Let's now hear from Ellen Aaronson and Brian Baker on behalf of the Hospital Library Caucus.

[Recorded message]

Donations to MLA by individuals demonstrate your commitment to support your colleagues with MLA grants, scholarships, fellowships, awards, and prizes. Those contributions help build MLA's endowment which, augmented by donations from foundations, institutions, sponsors, and MLA operations, provided close to $100,000 of support to140 individuals in 2021. Thank you to the 56 individuals whose combined gifts totaled $25,000 in calendar-year 2021. On behalf of the entire MLA Board of directors, we thank you for your generous support. We enjoyed making these thank yous! Capturing us all during the Zoom meeting was the biggest challenge—please know there were more grateful board members with wonderful signs that are not shown here.

This year has been an emotional rollercoaster. At my inaugural address, my final slide congratulated my mentor and friend Pat Gallagher on her retirement.

I was grateful to have the platform of my MLA presidency to acknowledge Pat's many contributions. Pat spent her precious time writing my MLA president biography that was published in the Journal of the Medical Library Association just a few days before she passed away.

This photo of Pat knitting at the 2018 MLA meeting in Atlanta brings back so many memories. Pat's husband, Steve Greenberg, has graciously donated many items knitted by Pat for a special online auction to benefit the MLA scholarship fund. We'll hear more about that in the future. For this moment, I just want to invite all of you to take a quiet moment to remember Pat.

[Pause]

Thank you all.

I knew the previous slide would be both wonderful and sad, and I would need something to make us smile. So, I invited a friendly bulldog to revisit my inaugural theme about health sciences librarians as unicorns. This is NOT my bulldog Bridget—she refuses to wear costumes—but she would probably be as cute.

I loved the idea of traveling and engaging with library professionals worldwide, especially our international affiliated associations and collaborations. It was disappointing that the pandemic meant I attended all of these meetings virtually. Virtual globetrotting brought tremendous joy even if it was very hard for me being in the far-left corner of this map in the Pacific Northwest to handle all of the time zones for the various meetings. Closest to home was the Canadian Health Libraries Association meeting at virtual meeting place in Winnipeg. Then moving on to the EAHIL virtual workshop in Istanbul, Turkey, and ending with the International Congress of Medical Librarianship and Association for Health Information and Libraries in Africa in March. My hope for the future is we fill more of this map by meeting with our colleagues in Central and South America as well as in Asia.

Offering a keynote on behalf of MLA at ICML/AHILA was the highlight of my year, and perhaps my career. I was honored to be part of this amazing group of colleagues. I owe special thanks to Dr. Grace Ajuwon of AHILA. We met at the EAHIL virtual workshop where each of us as leaders of our associations were asked to host coffee hours telling folks about our associations. That first connection led to further conversations and ultimately the chance to contribute as a speaker.

Dr. Grace is virtually attending the Medical Library Association meeting, so I hope you are able to meet her. I also had the pleasure of meeting our Cunningham Fellows Kubra at EAHIL and Mercy at ICML/AHILA and I cannot wait to meet them and our other Fellows in person at MLA 2023!

Now I'd like to review a few collaborations that have evolved and advanced towards more sustainable positions.

Librarians without Borders has been a tremendous partnership among MLA, the Elsevier Foundation, and Research4Life with many members and the International Cooperation caucus participating. At ICML, I heard the Elsevier Foundation and other speakers share about the new focus on country connectors, which aims to build local capacity and local user communities.

If you're interested in hearing more, there is a guest post in the Scholarly Kitchen from 2021 with more detail or visit the Elsevier Foundation website.

This summer I will collaborate with MLA's Mary Langman on clarifying and invigorating MLA's organizational affiliations. We partner globally with a wide range of both library and health associations. Caucuses and individual members often play critical roles in developing and maintaining these partnerships. We want to look at how appointed representatives, the MLA Board and headquarters staff work together to advance those partnerships. And we are open to collaborative opportunities that advance MLA strategic goals in advocating and educating on health information issues. If organizational affiliations are something that you're passionate about, please be in touch with me. We will be convening conversations among stakeholders and will share more information as we move forward.

Affiliated organizations are just a few of the important partners in amplifying MLA's voice on issues. In the area of publishing, within and beyond MLA there is a lot of potential to build on the work we did with the InSight Initiative discussed earlier.

The most tangible outcome for this year's annual meeting is the symposium on 21st Century Health Sciences Collection Development and Resource Sharing which was planned by a special program committee of caucus experts and vendor partners in part to follow on to opportunities suggested during InSight.

Thanks to a recommendation from the JMLA team, MLA signed on to C4DISC, the Coalition for diversity and inclusion in scholarly communications. We hope that this is a first step in engaging in advocacy with our publishing partners. We also recognize the growing Scholarly Communication Caucus, a wonderful place for members to continue the dialogue on knowledge dissemination.

In our final minutes together, I'd like to update you on three works in progress that I hope will take great strides in the coming months. Using Our Voice for MLA advocacy and partnerships is a major effort that we have to do together. Partner with your Hospital Librarian has a great starting group of signatories, but there are many more to pursue, and we have editorials and letters in mind to reach the audience of health care administrators in the venues they engage. At the April board meeting, we agreed with a proposal by Emily Brennan and Heather Holmes to collaborate with our Systematic Review Caucus and other organizations on a statement and strategy on recognizing librarian authorship for systematic reviews and guidelines. We are also thinking about our voice and our collective and individual roles in dismantling disinformation and misinformation—it is a crucial area that we highlighted during National Medical Librarians Month, and it needs partnerships as well as your ideas and collaboration.

In addition to our external relationships, we have a lot to do organizationally within MLA. Now that the Communities have been in place for several years, it is time to assess how the structure is working and what could improve to help Caucuses and Domain Hubs succeed. We have a great team led by Adela Justice, Chair of Community Council. Your voices are essential for this, so please engage with our surveys and request for interviews. After we report to the Board and to Community Council, we will share via MLA Connect.

We have heard the need to explore more sustainable approaches to producing this open, field-leading journal, which benefits our members and the community. Thank you to our interim editor in chief, Alex Carroll, and the amazing associated assistant editors who are leading during this transition. We hope to be able to announce soon how *JMLA* is going to advance in its next 3 years of publication. Stay tuned for that as the search committee for the editor or editors in chief moves forward.

On this final slide, I want to disclose the commitments that I made a year ago. I had really hoped and planned to finish the write up of my Kronick traveling fellowship before this meeting. Now the plan is to finish it for the 125th anniversary next year. What I contributed instead was working with an amazing team of researchers on the COVID Interlibrary Loan and Document Delivery multi-arm study. You can learn some of our findings here at MLA 22 and then also the survey findings in the July issue of JMLA. We again want to thank the more than 400 librarians who participated in that survey.

I recognize this year how we have all these tough choices to allocate our time. I want to acknowledge all of you who contributed to making your MLA, a wonderful community this year. To close, I'll paraphrase Beverly Murphy. I hope Beverly's here virtually, although I know she will not be at the meeting in person this year. We are all team MLA!

I welcome your questions and comments—please email me at my personal email address krisalpi@gmail.edu. Thank you.

### Conclusion

Kris Alpi: Thank you friends and colleagues for joining us today to kick-off MLA'22, “Reconnect, Renew, Reflect”. We look forward to spending time with you over the coming weeks for a variety of fun events and engaging learning sessions. Watch the meeting website for information and updates. We'll see you soon! The Opening Session is now closed!

Journal of the Medical Library Association 111 (1/2) January/April 2023 jmla.mlanet.org

## IN MEMORIAM

June 14, 2022, 1:00-2:00 p.m., central time (virtual)

Kris Alpi: Before we get started with awards and fellowships, MLA has a tradition to pause and recognize our cherished members who passed away in the last year. Their counsel and friendship will be deeply missed. We have produced this video you will see today to honor their memory. The MLA Fellows and MLA members have generously provided in-depth profiles and photographs for this year's in memoriam. We will share the In Memoriam on MLAnet. We encourage you to view it and share it with others to learn more and be inspired.

Lisa Traditi: Truly inspiring and beautiful. Thank you. Let's get started with our recognitions and awards. New this year, we asked our award recipients to record their names using “say my name”. To up our game even more, and to pronounce those names correctly, please look forward to the “radio” voice of MLA's very own Darell Schmick! Darell - thank you! We are so grateful.

## RECOGNITIONS AND AWARDS

June 14, 2022, 1:00-2:00P.M. Central time (virtual)

Kris Alpi: Donations to MLA by individuals, large and small, are a demonstration of your commitment to support MLA grants, scholarships, fellowships, awards, and prizes. Those contributions help build MLA's endowment which, augmented by donations from foundations, institutions, sponsors, and MLA operations, provided over $90,000 of support to 130 individuals in 2020. Thank you to the 57 individuals who gave over $20,000 in calendar-year 2020. We recognize them in the next 2 slides.

Lisa Traditi: We are fortunate and thankful to have talented members serve in various MLA editorial and coordinator positions. I would like to recognize them today.

- MLAConnect Editor: Christine Willis, AHIP
- JMLA Editor: Katherine Goold Akers
- MEDLIB-L Coordinator: Richard James
- Oral History Project Director: Carolyn Lipscomb, AHIP, FMLA

Thank you for all your efforts on behalf of the association. I will share more about Carolyn's contributions later in the program. MLA advances our mission through its programs and services. Those initiatives are successful thanks to the committed work of MLA volunteers in Caucuses, Committees, Juries, Task Forces, and as Allied Representatives. We recognize our volunteers for your expertise and time to advance your association and the profession.

Kris Alpi: This membership year, 235 new members have joined MLA. New members bring fresh ideas and energy and are essential to MLA's future and diversity. We will be welcoming new to MLA members and first-time conference attendees at our annual new member/attendee networking event, on Wednesday, May 12. This year's program features virtual speed networking and a keynote address by MLA Past President Beverly Murphy.

Lisa Traditi: The Credentialing Committee evaluates and grants membership to the Academy of Health Information Professionals, known as AHIP. There are 1,008 AHIP MEMBERS. This is an impressive 40% of MLA members who have earned the AHIP credential. Here are the names of new members in the Distinguished and Senior categories. Here are the names of the new members in the Member and Provisional categories. There were no new Emeritus members this year. Congratulations to all of you. Many MLA Chapters provide funding for their members to receive AHIP. Your support is essential. Thank you.

Kris Alpi: From coast to coast, including Alaska, Hawaii, and several Canadian provinces, MLA's 12 chapters serve as an important home for members. They offer regional meetings, grants and scholarships, and other programs at the local and state level. The MLA Board of Directors recently approved the merger of the New York – New Jersey and Philadelphia Regional Chapters into the NEW Liberty Chapter. For nearly three years, the Chapter Merger Steering Committee combed through each Chapter's structures to determine synergies and opportunities between both Chapters to ensure a smooth transition for everyone into one cohesive group.

Lisa Traditi: Now I have the pleasure to present MLA's Chapter Project of the Year Award which recognizes excellence, innovation, and contributions to the profession of health sciences librarianship. This year's recipient is the Northern California and Nevada Medical Library Group for its goals of promoting diversity in health sciences librarianship in a meaningful and sustainable way. Let's hear a few words from the chapter past-president, Rachel Keiko Stark, AHIP, NCNMLG Chapter.

Kris Alpi: Enabling our members to pursue their professional Development regardless of their financial ability is an important MLA inclusion and equity objective. This year, we have so far provided 50 no-charge MLA'21 vConference registration to members. The $17,500 funding for this initiative comes from the proceeds of the Ysabel Bertolucci MLA Annual Meeting Grant and EBSCO/MLA Annual Meeting Grants endowments, augmented by MLA's operating budget.

Lisa Traditi: MLA's professional Development program is the hallmark of our association. MLA grants offer members opportunities to pursue professional Development courses that promote excellence in the field of health sciences librarianship. MLA offers several funding opportunities to assist qualified students in graduate library science programs. This enables members to further their careers and brand themselves as experts in their organizations. Here's Darell to announce the award recipients.

Darell Schmick: The Naomi C. Broering Latinx Heritage Grant provides a librarian with an interest in Latinx community information services the opportunity to pursue a professional activity in cutting-edge medical information services using the latest technical formats. This year's recipient is Hanni Nabahe. The grant will allow Hanni to promote a community-academic partnership that minimizes information silos in services critical to essential workers within the Spanish-speaking community and who have been particularly affected by the COVID-19 pandemic. The Continuing Education Grant supports continuing education to develop applicants' knowledge of the theoretical, administrative, or technical aspects of librarianship. This year there are two recipients. Alanna Campbell will participate in the Service Design: Towards a Holistic Assessment of Library Services course, where she will learn about the various tools libraries and librarians can use to implement a service design approach to assessment. Alessia Zanin-Yost, AHIP plans to advance her professional skills and gain new ideas on how to support students and faculty by participating in a series of CE courses. The MLA scholarships are awarded to students enrolled in or entering an ALA-accredited library school. This year's recipients are:

- Claudio Garcia, shared that his interests in scholarly communication and open access have deepened as he's had the opportunity both in his job and coursework to explore the ways that the research ecosystem impacts real-world healthcare outcomes. Claudio wants to become a health sciences librarian so he can help people live healthier lives by connecting clinicians and consumers to actionable, quality, health information.
- Laurier Lynette Cress has a professional objective to make the resources and materials within an institution as equitable as possible. This includes impartial and accurate metadata curation, designing and implementing an acquisitions policy built upon diversity and inclusion, and promoting the institution's collections to as many people as possible.

Kris Alpi: Thank you Darell. Now on to our international awards. MLA seeks to establish global partnerships and support the information needs of underserved individuals throughout the world. Today we are pleased to recognize members and colleagues who strive to educate and network with practitioners around the world. Their work makes a difference by advocating for access to information and helping to set standards for health information management.

Darell Schmick: The purpose of the Cunningham Memorial International Fellowship is to assist in the education and training of health science librarians from countries outside the United States and Canada. This year's recipient, Mercy Wamunyima Monde, looks forward to traveling next year. She plans to use the fellowship to enhance her leadership capabilities in health science librarianship in Zambia while sharpening her systematic review skills, Evidence Based Medicine skills, consumer health information skills and knowledge translation skills. MLA members will have an opportunity to meet Mercy in-person next year when we all gather in New Orleans. Let's continue our world travels. The MLA Librarians without Borders®/Elsevier Foundation/Research4Life Grants are an expansion of the Elsevier Foundation Librarians Without Borders/E-library Training Initiative. This program supports HINARI/Research for Life training activities that promote the use of the scientific resources in emerging low-income countries. Five institutions received 2019-2020 grants. The first four are:

- Sir Albert Cook Library, Makerere University, Kampala, Uganda
- Nelson Mandela African Institution of Science and Technology Library, Arusha, Tanzania
- Egerton University, Kenya, and
- Craper Benin, Centre de Recherche Agricole et Promotion des Expertises Rurales, Montreal, QC, Canada.

The fifth recipient is a joint team from:

- Medical Library, Children's National Medical Center, Washington, DC, and Bahir Dar University, Bahir Dar, Ethiopia.

Congratulations to all of the Librarians Without Borders grant recipients. The T. Mark Hodges International Service Award honors an outstanding individual achievement in promoting, enabling, and/or delivering improvements in the quality of health information internationally. The 2021 award is presented to Bethany S. McGowan, AHIP.

Lisa Traditi: I always appreciate hearing from our colleagues around the world. Next, our achievement awards. Time and time again, MLA members demonstrate what it means to be an outstanding practicing health sciences librarian or library educator. Today, we honor those that exhibit significant excellence in the areas of teaching, mentoring, and volunteering at the local, regional, and national levels. Here is Darell with the Achievement award recipients.

Darell Schmick: The Virginia L. and William K. Beatty Volunteer Service Award recognizes a medical librarian who has demonstrated outstanding and sustained service to the Medical Library Association and the health sciences library profession. The 2021 recipient is Nancy Schaefer, AHIP. Her volunteer spirit and drive to help others and uplift the profession are apparent in all of her MLA work, particularly within the Public Health/Health Administration Caucus. Nancy is the behind-the-scenes leader across MLA, contributing her expertise, her passion, and her knowledge to further MLA's goals. The Estelle Brodman Award for the Academic Medical Librarian of the Year is given to a member who has made outstanding contributions to academic medical librarianship. I am pleased to share this video from Karen Gutzman, the 2021 recipient. The Lois Ann Colaianni Award for Excellence and Achievement in Hospital Librarianship is given to a member who has made significant contributions to the profession through overall distinction in hospital librarianship. Let's hear from this year's recipient Nancy A. Clark. The Consumer Health Librarian of the Year Award recognizes a consumer health librarian who exemplifies the best in consumer health librarianship. We are pleased to present the 2021 award to Shari Clifton. A tenured Professor with more than 20 years of expert experience in health science, Clifton has provided significant consumer health information training in a variety of focus areas and to diverse consumer populations. This training has extended to public librarians and fellow colleagues, as well as students. Clifton has also developed a training program for public librarians and others seeking to obtain the MLA Consumer Health Information Specialization. The Louise Darling Medal for Distinguished Achievement in Collection Development in the Health Sciences recognizes accomplishment in collection Development for the health sciences. This year's winner is the Collection Development Caucus of MLA. The Collection Development Best Practices project is a noteworthy endeavor in both its aim and design that assists fellow librarians in acquiring or enhancing knowledge about the responsibilities and tasks involved in health sciences collection Development. The Carla J. Funk Governmental Relations Award recognizes a medical librarian who has demonstrated outstanding leadership in the area of governmental relations at the federal, state, or local level and who has furthered the goal of providing quality information for improved health. Barbara A. Epstein, AHIP, FMLA is the 2021 recipient. The Lucretia W. McClure Excellence in Education Award honors outstanding practicing librarians or library educators in the field of health sciences librarianship and informatics. Let's hear from the 2021 recipient Jodi L. Philbrick, AHIP.

Kris Alpi: It's inspiring to hear the impact that MLA members are making in the field of health information sciences. The Rising Star Program began as a biennial initiative in 2010-2011 sponsored by the Board of Directors. It has now grown into an annual leadership Development program that matches each Rising Star with a mentor in a comprehensive curriculum that includes monthly classes, training to develop self-awareness of skills, and a group project that relates to current MLA initiatives. Let's hear about this year's outgoing Rising Star class and the incoming one.

Darell Schmick: The MLA Rising Star program gives these members the opportunity to develop skills and knowledge needed to become a leader in MLA. The 2020-2021 cohort of Rising Stars has been quite busy this past year. You are all invited to join them for their presentation on June 23, 2021, where they will share their research “Advocacy is All of Us: Recommendations to Enhance MLA's Advocacy Initiatives” I am pleased to introduce you to the ninth Rising Stars cohort. We look forward to seeing and hearing more from our new stars. You will have an opportunity to meet them in-person and learn about their experience next year when they present their work at MLA ‘22 in New Orleans.

Lisa Traditi: Thank you to the many members that volunteer their time and efforts cultivating, mentoring, and directing the Rising Star program. Each year MLA recognizes both published and unpublished works that highlight health sciences librarianship and the many innovations taking place in the field. These works often have a significant impact on librarians in different settings such as academics, hospital, and special libraries. And the winners are…

Darell Schmick: The Ida and George Eliot Prize is awarded for a published work that has been judged most effective in furthering medical librarianship. The winning article is: “Evaluating nursing faculty's approach to information literacy instruction: a multi-institutional study,” Let's hear more from team member Bethany McGowan. The Erich Meyerhoff Prize was established to recognize and stimulate health sciences librarians' interest in the history of medicine. The 2021 prize is awarded to Aidy Weeks, AHIP. Her manuscript offers a fresh perspective on an important figure in the history of the MLA through an investigation of racial bias in several of Mary Louise Marshall's published works. The methods for identifying the sources selected for review are sound and transparent. The arguments set forth are astute and persuasive. The subject matter is relevant and timely, given the current state of American society, the current state of the library profession, and MLA's efforts to emphasize diversity, equity, and inclusion over the past several years. The Rittenhouse Award recognizes the best unpublished paper on medical librarianship. Robert Browder is this year's recipient for his paper on the potential of blockchain technology to change the way health information is shared among healthcare providers and organizations. This has the potential to be very impactful in the field of medical informatics, as well as patient health, and invites discourse on the implications of blockchain technology on transparent communication, privacy, and security.

Kris Alpi: Congratulations to all of you. One of MLA's professional competency areas is the ability for health sciences librarians to understand scientific research methods and to critically examine and filter research literature from many related disciplines. MLA supports members in their research endeavors and recognizes those that advance evidence-based practice. Today, we acknowledge members who have made significant contributions and challenge us all to use the best available evidence. Here are the recipients of MLA research awards.

Darell Schmick: The Donald A.B. Lindberg Research Fellowship funds research linking the information services provided by librarians to improved health care and is awarded to a qualified health professional, researcher, educator, administrator, or librarian. The 2021 Lindberg Fellowship is awarded to Brandon Patterson and Kerri Shaffer. Their research “Implicit Bias in Health Care: Exploring the Upstream and Downstream Effects with Virtual Reality,” will explore the cultural roots of bias and how that bias impacts treatment provided by health care providers. Specifically, the project aims to better understand implicit bias in hopes of addressing and affecting health care providers behaviors. The project uses Virtual Reality to observe and track data points in simulated health care providers-patient interactions. Branson Patterson is a key staff member of the sponsoring institution's library and its Virtual Reality Lab. The Research Advancement in Health Sciences Librarianship Award recognizes organizations whose exemplary actions have served to advance health information research and evidence-based practice in health sciences libraries. We are so pleased to have two recipients this year. The MLA Research, Development, and Demonstration Project Grant supports projects that will promote excellence in the field of health sciences librarianship and information sciences. This team proposes to promote excellence in health sciences librarianship and more effective interdisciplinary collaboration between librarians and nurses in both clinical and academic settings with a qualitative research study. The overall goal is to raise awareness for nursing faculty, nursing students, and practicing nurses regarding the need to evaluate resources based on a variety of criteria.

Lisa Traditi: It is now my honor to share with you the recipients of some of MLA's most prestigious awards. Fellows of the Medical Library Association are chosen for their outstanding contributions to health sciences librarianship and to the advancement of MLA's purposes. This year the Board of Directors has named three association members as MLA Fellows. MLA Fellows - Linda Walton, AHIP, FMLA, is the Associate University Librarian at the University of Iowa. Her outstanding and groundbreaking career in health sciences librarianship has spanned over 40 years. Her contributions to health sciences librarianship, both nationally and internationally, are numerous and significant. Linda's extensive record of service to MLA includes serving on MLA's Board of Directors as Treasurer (2007-2009) and MLA President (2014-2015), Known as a down-to-earth, compassionate, and supportive person, she has mentored dozens of librarians over the years, encouraging them to step outside of their comfort zone to do interesting and meaningful work. Linda is much admired by her MLA colleagues. Kristine M. Alpi, AHIP, FMLA, has had wide-ranging and positive influence on the Medical Library Association. Her impact in health sciences librarianship is broadly felt through her research and scholarship. One of Kris's most distinct attributes is her ability to turn questions that come up in normal library operations into excellent research questions that end up contributing to health sciences libraries. She is known as a compassionate leader who always strives to keep her staff and patrons at the forefront of her vision. Janna C. Lawrence, AHIP, FMLA, has made significant, sustained contributions to MLA through her leadership and the investment of her talents to advance the association. Her great enthusiasm is evident, as she welcomes new members to MLA or through the mentoring of new/young members, which she has done quietly throughout her career. In her role as 2020 NPC co-chair, Janna displayed great patience and tact while managing member expectations during the shift to the 2020 all-virtual conference. Kris, Janna, and Linda now have “FMLA” as an added credential right next to their AHIP. Congratulations to you all and thank you for all you do for MLA and the profession.

Lisa Traditi: Each year, the MLA President has the honor of recognizing librarians who have enhanced the profession of health sciences librarianship and furthered the objectives of the association. Recipients of the President's Award will all receive a unique MLA lapel pin. When we are together in-person or on video in the future, I hope they will wear their pins proudly. Now, it is my privilege to present the MLA President's Award for 2021. Since 2009, Carolyn E. Lipscomb, AHIP, FMLA, served as Program Director of MLA's Oral History Project. Under her leadership, the project saw an organized workflow that resulted in smooth and consistent interviewing, editing, and publishing. Carolyn oversaw 27 interviews, published 43 oral histories, and introduced the advent of several digital publications that are available on MLANET. On behalf of the Board and MLA members, we thank Carolyn for her work to preserve the interviews that help illuminate the history of health sciences librarianship and the association. The authors of MLA's COVID-19 Resource pages and those of the Spanish-language COVID-19 Resources responded in record time to create an invaluable resource for librarians, our communities, and health care consumers. Thank you to all of you for such an important work. Your work highlights the value of hospital and clinical librarians to the association and the communities they serve. Thank you for your work to provide access to reliable Covid-19 information in Spanish. Diversity and Inclusion became a strategic goal of MLA in 2017. President Barbara Epstein established the Diversity and Inclusion Task Force in August of 2017. This team presented their final report to MLA in 2020, with a call to create a permanent Diversity, Equity, and Inclusion Committee. Please take a moment to read the names of all your colleagues who were part of this initial task force. Task Force chair, Sandra Franklin, now joins us to accept the award.

Kris Alpi: This next distinction is very special. The Marcia C. Noyes Award is the highest honor that MLA confers on any individual. The individual also gets the traditional silver bowl from MLA, and the beautiful flowers from the Foundation of MedChi, the Maryland State Medical Society, where Marcia C. Noyes worked and lived for 50 years. Here to introduce this year's recipient, as is our tradition, let's hear from the 2020 Marcia C. Noyes Award recipient, Gerald J. Perry, AHIP, FMLA.

Lisa Traditi: And now, live from Durham, North Carolina, please welcome Beverly Murphy, AHIP, FMLA, this year's Marcia C. Noyes Award recipient. Hi Beverly. So great you could join us today!

Beverly Murphy: [Live acceptance talk]

Lisa Traditi: Congratulations, Beverly. You are an inspiration to us all. What a wonderful way to finish the awards session.

Kris Alpi: The awards presentations remind us of the outstanding accomplishments our peers make to the profession of health sciences librarianship. It also encourages us to continue to grow towards new levels of achievement. Congratulations everyone!

Lisa Traditi: Thank you friends and colleagues for joining us today to kick-off the 2021 vConference. We look forward to spending time with you over the next three weeks for a variety of fun events and engaging sessions. We'll see you soon! The Opening Session is now closed!

[End of opening session]

## MLA '22 ANNUAL BUSINESS MEETING AND INCOMING PRESIDENTIAL INAUGURAL ADDRESS (PRESENTED VIRTUALLY)

Thursday, May 12, 2022, 1:00-2:00 p.m., central time

MLA Board Members

- Kristine M. Alpi, AHIP, FMLA
- Kevin Baliozian
- Donna R. Berryman, AHIP
- Sally Gore
- Heather N. Holmes, AHIP
- Shannon D. Jones, AHIP
- Adela V. Justice, AHIP
- Janna C. Lawrence, AHIP
- Brenda M. Linares, AHIP
- J. Dale Prince, AHIP
- Meredith I. Solomon, AHIP
- Lisa K. Traditi, AHIP
- Tara Douglas-Williams, AHIP

MLA parliamentarian

- Chris Shaffer, AHIP

MLA sergeant-at-arms

- Linné Girouard, AHIP

Incoming MLA Board members

- Amy Blevins, AHIP
- Emily Hurst, AHIP
- Tony Nguyen, AHIP
- PJ Grier

Others

- Mary Langman
- Maria Lopez
- Debra Cavanaugh
- James Westwood
- Kate E. Corcoran

### Welcome

Kris Alpi: Greetings! I am Kris Alpi, your MLA President. I am pleased to officially welcome you to the 122nd Annual Business Meeting of the Medical Library Association, over which I will be presiding as my final Presidential act. Last year, more than 500 members attended our second virtual business meeting, continuing to surpass our previous attendance records from in-person business meetings. We are thrilled that so many of you attended and that being virtual is helping us be so much more inclusive. It is wonderful that so many of you are participating with us today. Welcome!

We have a simple agenda, with lots of information we hope you find compelling. The MLA Annual Business Meeting includes:

- the presentation of your current MLA Board of Directors
- reports by the MLA Treasurer and Executive Director
- election results and the presentation of your new MLA Board

Then, we will hear from our Incoming MLA President, Shannon Jones, AHIP, FMLA, who will present her Inaugural Address.

Before we get started, here are a few guidelines:

- All MLA gatherings and interactions need to respect MLA's Code of Appropriate Conduct. Please consult it online. If you need to report a violation, there is a link to do so on the web page.
- We are using Zoom webinar today, with which, by now, you are all likely extremely familiar.
- We will be using Zoom polls for voting, as well as the “raise your hand” feature” for official business. We'll walk you through procedures in a few minutes.
- We will NOT be using the Q&A feature today. Parliamentarian Chris Shaffer will share the formal business meeting process for raising an issue soon. During the year, we offer topical open forums on many areas of MLA to invite conversations and questions and offer a better experience for dialog.
- Feel free to use the chat, but please note that we will NOT be monitoring the chat for questions. If you have a question that you would like addressed after the meeting, please email president@mail.mlahq.org

To get us started with the Annual Business Meeting, I would like to recognize Chris Shaffer, MLA's parliamentarian. Chris will assist us with the business portion of our meeting.

Chris Shaffer: Hello fellow MLA members. This is our third electronic business meeting, so we are getting to be pros at this. Before I get started in my official role, let's practice some of the Zoom Webinar features to make sure you are familiar with them before the real deal.

First, let's practice hand raising. During the meeting, there will be times when you will have the opportunity to raise your hand to speak to an issue on the floor in the context of the official business of this meeting.

Here is the fine print:

- A written motion is introduced
- The presiding officer restates the motion and asks if there is discussion
- Members wishing to address the motion can “ raise their hand”
- Staff will let the presiding officer know you wish to address the motion
- The presiding officer gives you permission to speak for up to two minutes
- Please announce yourself before you ask your questions or raise an objection
- This process is repeated until all who have raised their hands have had two minutes to speak to the motion on the floor
- Anyone wishing to speak a second time may raise their hand and have one additional minute to speak
- The presiding officer will then repeat the motion and call for the vote

As to the specifics on how to “Raise Hand” and speak in Zoom Webinar:

- We will monitor who has raised their hand, share this information with the President, who will then call on you by inviting you to speak.
- You will see a button appear on your screen prompting you to turn your microphone on. You may optionally turn on your video as well.
- Once you do, please announce your name, and speak.
- Once you have spoken, please lower your hand.

Please locate the “Raise Your Hand” button on your Zoom control bar. If you press it, please also unpress it so that all hands are lowered when we get started.

Now let's go through the specifics of voting: when the presiding officer calls for a vote on a motion:

- You will see a poll appear on your screen
- Vote “Yes”, “No” or “Abstain”. Don't forget to press the “submit” button.
- You have two minutes to vote.
- After the vote, the results will appear on the screen, and will also be announced by the Sergeant-at-Arms.

Now, let's practice. A poll will appear on your screen. Go ahead and vote. We'll keep this to a short 30 seconds for this practice session. And here are the results of the vote. Well done. So now that you know how to raise your hand and how to vote, let me share with you that you may not actually have to do that today.

Robert's Rules of Order allows business to be conducted by unanimous consent, which removes the need for discussion and a full vote. Any member present may object to unanimous consent and require the President to open the floor for discussion and put the question to the members for a vote. Today, we plan to use unanimous consent and will use the “Raise your Hand” feature to allow members to register an objection. All new business must be presented by a member in the form of a written motion and submitted to the President. As announced on April 22nd, members are strongly encouraged to submit motions in advance of the meeting.

### Quorum Count and Call Meeting to Order

Kris Alpi: Thank you, Chris. I'm pleased to introduce Linné Girouard, AHIP, MLA's Sergeant-at-Arms, who will assist us with the counting of the quorum.

Linne Girouard: Hello everyone. Great to be back for my annual official business meeting duty! As Sergeant-at-Arms, one of my roles is to monitor login counts and names of member participants, to ensure we have a proper quorum. As voting members of MLA, you were provided the link to this session, and asked to type in your name and email address to enter the meeting. According to MLA Bylaws a quorum of 200 of the voting members is required for transaction of business. Madame President, there are 251 members present.

Kris Alpi: There being more than 200 voting members present, we have a quorum. I now call our meeting to order.

### Presentation of the Agenda

Kris Alpi: I would now like to welcome Heather Holmes, MLA's Secretary. Hi, Heather.

Heather Holmes: Hello Kris and fellow MLA members. Great to be joining you today. As MLA secretary, I get to review Board minutes. I also get to present the agenda for the 2022 business meeting which you can now see on your screen, and which is available on MLANET. We've done the first three items already.

Kris Alpi: Thank you, Chris, Linné, and Heather. Please stick around as we may need to call on you.

### Presentation of the 2021-2022 Board of Directors

Kris Alpi: Now, please welcome Kevin Baliozian, MLA's Executive Director. Hello, Kevin.

Kevin Baliozian: Hi, Kris. Great to be here. I have the honor to introduce the 2021-2022 MLA Board of Directors. We are so fortunate to have such a strong group of leaders. This Board of Directors has expertly steered us through a second year of the pandemic, along with other issues of global impact. Thank you.

Introducing the members of MLA's 2021/22 Board of Directors:

- President: Kristine Alpi, AHIP, FMLA
- President-Elect: Shannon Jones, AHIP, FMLA
- Immediate Past-President: Lisa Traditi, AHIP
- Treasurer: J. Dale Prince. AHIP
- Secretary: Heather Holmes, AHIP
- Chapter Council Chair: Donna Berryman, AHIP
- Community Council Chair: Adela Justice. AHIP
- Directors:
  - Sally Gore
  - Janna Lawrence, AHIP, FMLA
  - Meredith Solomon, AHIP
  - Brenda Linares, AHIP
  - Tara Douglas-Williams, AHIP

Please congratulate your 2021/22 Board of Directors and see if you are able to match the names to the photos!

### Treasurer's Report

Kris Alpi: Thank you, Kevin. Now, please welcome J. Dale Prince, your MLA Treasurer, who will spend the next few minutes updating us on our finances. Hi Dale - welcome to our business meeting. It's always good to hear from you.

Dale Prince: Thank you, Kris. Hello everybody. As your treasurer, I share the financial stewardship of our association with Kevin, your Executive Director, and rely on the insights and review of the Finance Committee to ensure the Board of Directors can exercise its duty of care.

The finance committee was very busy this past year, and a little stressed out too.

In addition to:

- reviewing the budgets and financials prepared by the MLA staff,
- working with the MLA independent auditors to ensure compliance and best practice,
- setting MLA's investments strategy with MLA's independent financial advisor,
- examining key MLA pricing models, and
- analyzing contract terms with MLA's management company, MCI USA
- we had to ensure the continued financial sustainability of MLA during Covid-19
- New this year: the finance committee reviewed and approved funding requests from caucuses and domain hubs

I am so grateful to have been supported in my role as treasurer by such an experienced group of colleagues.

Please take some time to thank the members of the finance committee:

- Lisa Traditi, as Immediate Past President
- Donna Berryman, as a member of the Board
- Teresa Knott, as a MLA member at-large
- Janna Lawrence as a member of the Board
- Deborah Lauseng as an MLA member at-large
- Kevin Baliozian, as your Executive Director
- Kristie Hammill, as your MLA Director of Finance

Let's start by reviewing financial numbers.

- 2019 was the last year prior to Covid-19, so I thought it useful to include it for comparative purposes with our 2020 and 2021 Covid-disrupted years.
- Both 2019 and 2020 financial years have been audited.
- The 2021 numbers are pre-audit, so may be adjusted by MLA's audit firm.

The top three lines are what we refer to as “operating”: that includes all financial activities except investment revenues and disbursements from the MLA endowment for awards and grants.

- Revenues have steadily decreased. In the next slide, we will see that the true decrease of revenues is higher than the $464,000 from 2019 to 2021.
- Though expenses have also decreased, they haven't done so to the same level as revenues.
- That in turn, increases the operating net margin, which is a loss for all three years.
- The non-operating net margin is the difference between the financial revenue of our endowment and reserves and the disbursements from the endowment fund. As you can see, MLA has had excellent performance.
- As of the beginning of 2022, the stock market performance has been dismal, so we do expect from time to time to have a negative return. This being said, at this time in 2020 the market was significantly down, and more than recovered by the end of the year. Time will tell.
- When you add up operational with non-operational, you get to the net change in assets, which has been positive all three years.
- This means that MLA's net assets have kept growing, even through Covid.

The prior slide was the accounting view, based on applying general accounting principles.

For a clearer understanding of MLA's operating performance, we have adjusted the numbers to represent an adjusted EBIDTA, also known as Earnings Before Interest, Depreciation, Taxes, and Amortization.

The adjustments include the list on the right side of the graph, which are important to note:

- We excluded the investment revenue. It's real, but it doesn't reflect operations.
- We excluded the investment in EFTS and the associated depreciation. This is a technical adjustment to reflect EBIDTA. Note that we invested $157,000 in the technology platform, fully funded by participating libraries.
- Same for the investment in education. Note that we invested $191,000 in new education programs, as part of MLA's strategic initiative.
- MLA received $233,000 in PPP money from the CARES act, which was forgiven. That is actual money that we will NOT have to pay back and designed to support associations in our situation. We are grateful for it.
- We removed an additional $43,000 in miscellaneous investments.

Take a look at the graph:

- In 2018, we were balanced.
- In 2019, we had a $175,000 deficit, which we had intended to address over the following years. Then Covid hit.
- 2020 was the first year of Covid, with obvious financial damage, mostly linked to the cancellation of the in-person conference.
- In 2021, the Covid negative effect was even higher: Exhibitors did not value the virtual conference, so pulled back in year 2.
- Our 2022 budget shows an improvement, though many uncertainties remain. Covid continues to have a negative impact on our 2022 finances.

The financial resilience of MLA is related to our net assets. Those represent the cumulative net revenues and losses over the years. The $4 million is an impressive number, split between the endowment in orange and reserves in blue. An increase or decrease in the value of our investment directly affects that number.

So, what's the bottom line:

- We have a structural operational deficit that is the result of overdependence on membership revenue and the annual conference. We have a long-term plan to address this deficit and have sufficient reserves to achieve this objective without limiting our services nor investments.
- Our net assets went up because the stock market did well, and because we received government support.
- Our cash position is very strong. We also applied for and received a $500,000 EIDL loan at a very low interest rate. That increases our flexibility. We can reimburse it at any time or over the 25 years of its term.

In summary, I am happy to report that the state of MLA finances is strong. Because the MLA annual conference has such a significant impact on MLA finances, I wanted to share with you numbers for the last few years.

On the left, the attendee registration counts:

- In blue, the in-person registrations.
- In orange, the virtual-only registrations.
- Our total attendance is up for all 4 years, as a result of the virtual component, and the quality of the programing.

On the right, here are the financial revenue numbers of the conference:

- In blue, the registration numbers.
- Though registration counts are up, registration revenue is down.
- That's a problem because the cost of running a virtual conference is as high as running an in-person conference. A hybrid conference is even higher.
- In orange, revenues from exhibitors and sponsors. As you can see, those are correlated to the in-person attendance.

Our challenge is to adjust the MLA conference and pricing model to sustain hybrid AND the profitability requirements that sustain MLA.

The Finance Committee took on the responsibility to review and approve funding requests from caucuses and domain hubs. In the last 15 months, 10 requests were approved out of 12 submitted, for a total of $6,642. The evaluation criteria are listed on the slide. Our guidelines include:

- Ensuring requests are aligned with mission.
- Favoring initiatives that benefit the whole group vs. the individual.
- Making sure that if there is a better way to achieve the outcome, we direct the individual or group to that path. We want to make sure we are good stewards of MLA funds.

Starting in June, the finance committee will include representatives from Community Council so that our group can better understand the context of the request. Note that the funding for awards, scholarships, prizes and travel grants is now funded from the MLA endowment or operations, rather than by funding requests. Same goes for the annual conference, where we now have group social activities funded by the MLA conference budget.

That does it for the Treasurer's Report.

Kris Alpi: Thank you, Dale. The board is grateful for your diligent stewardship of the entire financial team.

### Executive Director's Report

Kris Alpi: The next order of business is the Executive Director's report. Kevin, welcome back.

Kevin Baliozian: Thank you, Kris and thank you Dale for your financial summary and outlook. Let's start by reviewing the current MLA strategic goals.

The Board launched the Building a Better Future goal last year. The objective is to both look back at our 125-year history, and build a better future, with particular attention on being inclusive of all communities. The Board established 3 task forces, one for each of the strategies:

1. Honoring 125 years of history with publications, events, and initiatives that highlight MLA milestones, deepen our understanding of the experience of diverse communities over the course of MLA history, and ensures that all voices are represented within the historical narrative,
2. Envisioning the future (25 years) of the profession with community-driven activities and ideation that reflects the richness of MLA communities,
3. Creating an online repository of MLA historical intellectual property and fundraising for the initiative.

This second Education goal follows a first Education goal started in 2015. We have a long-term plan to:

- position MLA as the go-to education resource for health information professionals
- foster excellence in the professional practice and leadership of health sciences library and information professionals
- grow revenue outside of membership and the annual conference

The Communities strategic goal is also the continuation of the Communities Transformation goal started in 2016. It is ambitious and at the core of our We Are MLA vision. Success will be achieved when we successfully

- increase the relevance, effectiveness, and value of MLA,
- ensure that all health information professionals at all career stages can find a professional home within MLA,
- empower MLA members at the grassroots level,
- increase member engagement,
- improve the quality of MLA programs,
- attract more members, and
- reach audiences beyond members.

The last strategic goal is to Reinvent the Meeting Experience. This goes beyond the annual conference and has been particularly relevant because of Covid-19. We are well on our way to achieve our objectives to:

1. develop new ways of combining virtual and in-person models (“hybrid”) for the MLA annual conference and other events,
2. integrate technology and instructional design that engages the attendee,
3. develop new business models for attendees (members and non-members) and vendors,
4. collaborate with MLA Chapters and other local stakeholders, and
5. engage with members and non-members throughout the year.

Experience MLA is a wonderful example of a successful initiative that achieves objectives of both the Communities and Meeting Experience goals.

The MLA Diversity, Equity, and Inclusion goal that “aimed to evaluate and improve MLA practices as they relate to diversity and inclusion within MLA” was completed in 2020. The MLA DEI committee was established, and the decision was made to explicitly include DEI objectives and measures in everything we do, rather than address DEI as a separate goal.

The 2021-2022 MLA Annual Report will be presented in the next segment. One of those reports is the Headquarters Report, and I invite all of you to read it, as it contains great information.

In this report you will find about facts such as:

- NLM appropriation numbers, and MLA efforts to support the NIH and IMLS, and a lot more.
- Submissions and percent acceptances for the MLA'21 and MLA'22 contributed content. There were 372 in 2022 up from 325 in 2021.
- 199 of you applied for committees in 2022. All will be appointed, and we will be communicating with you by the end of May.
- Caucus members numbers since 2018. The Social Justice and Health Disparities caucus had 39 members in 2018 and now has 267.
- MLA offered 21 webinars and 8 instructor-led courses, and now has 19 self-paced courses. We also have launched the Data Services Specializations and the Systematic Review Services Specialization.
- There are 951 current AHIP members.
- In 2021, $1,702,240 of funds were exchanged between the 879 members of the MLA EFTS platform. That's 146,623 ILL transactions including those at no-charge.
- The JMLA impact factor is up and now an impressive and all-time high 3.18
- Traffic on the MLANET website was 30% higher in 2021 than in 2020, and double what it was in 2019.
- Find out what books MLA approved to be published, and statistics on InSight, the RTI and more.

Experience MLA provides a 4-week free membership to experience what MLA has to offer. Caucuses drive the activities by organizing events, meet-ups and other activities.

We launched Experience MLA in 2021 and saw a significant increase in participation by caucuses and individuals in 2022.

In February 2022:

- 35 caucuses and committees participated
- 1,600 individuals joined activities, including 202 individuals new to MLA

Regarding MLA membership, we are delighted that for 2022, we have stopped the MLA membership decrease of the last 10+ years. On April 30, we have 2,350 members and 84 more than at the same time last year. Our retention rate is slightly higher than in 2021, and the main factor is more than a hundred more new and rejoining members in 2022 than this time last year. Thank you to our 356 new and rejoining members!

Other impressive data is that on average, a member who participates in a caucus will participate on average in four caucuses. That's up from under three prior to the community transformation. Eliminating the cost barrier to communities has had a measurable effect on participation counts.

So many of you have been a part of transforming the voice of MLA, by making it more inclusive, diverse, and authentic. Thank you.

Your voice expresses itself in multiple ways, through:

- The We Are MLA project. Please read the profiles by clicking the link on the homepage.
- The voice of caucuses. Caucuses today issue their own statements on issues of significance to their constituencies. Those are also accessible through the homepage.
- I invite all of you to stay informed about the history project. It's part of our strategic goal and will be part of the 125-year MLA celebration starting in 2024.
- The MLA book panel now reviews DEI objectives in book applications.
- More on JMLA in the next slide.

Thank you to the DEI committee for continuing to guide all of us in walking the walk, in diversity, equity, and inclusion.

Taking a deeper look at the voice of MLA from the MLA history perspective, our objective is to deepen our understanding of the experience of diverse communities over the course of MLA history and ensure that all voices are represented within the historical narrative.

The areas to address, such as implicit and explicit bias and under-representation are identified in the left column.

Specific solutions include:

- reviewing and expanding the criteria for how interviewees are selected to increase the number of histories from BIPOC librarians and those from other underrepresented groups.
- inviting members from MLA's identity caucuses including the Accessibility and Disability Caucus, African American Medical Librarians Alliance, Latinx, LGBTQIA+, and Asian Diaspora Coalition to join the conversation and work with the committee.
- reviewing the program structure and possibly creating an editorial board to oversee the program.
- identifying training opportunities for members interesting in conducting, editing, and publishing oral histories.

In late 2020, when BIPOC authors were invited to publish an editorial on anti-Blackness in librarianship, they received pushback when expressing themselves in their submitted piece due to a rigid editorial process.

This crisis accelerated the efforts to change JMLA practices and culture. As a result, JMLA:

- Now has an open call for editorial board members rather than the editor-in-chief identifying new members. While all members continue to be selected based on their publishing and peer review experience, the open call has led to more editors from underrepresented groups. We welcomed:
- Thane Chambers as an assistant editor
- Beverly Murphy, AHIP, FMLA and Tashiana Scott-Cochran are our new obituaries editors
- Matthew Noe as the social media editor
- There are calls for reviewers with expertise in DEI issues and critical theory
- JMLA is exploring a new editorial internship program to provide health sciences librarians who are new to scholarly publishing, particularly those from underrepresented groups, with mentored peer review training and an insider view of journal editing.
- Giving authors agency over their work from the beginning to end of publication process:
- At submission: Authors can recommend peer reviewers who are particularly well suited to comment on their manuscript.
- During revisions: authors are not required to make every change recommended by a reviewer or accept edits that change the meaning of the text or alter ideas they wish to convey.
- After acceptance: authors receive a word doc with tracked copyedits/changes.
- A workgroup that was created and tasked with reviewing the MLA's Style Manual. Expected changes include:
- making the manual more gender inclusive and
- adding a new section with guidance on preserving authentic voice and honoring the author's word choices.
- Another positive change surrounds creating a pipeline of diverse content for MLA annual meetings. A template has been created that members can use themselves help bring in new librarian colleagues and those from underrepresented groups to submit Papers and posters for MLA conferences.

The JMLA board is now more diverse, better represents JMLA's broad readership and is better prepared than before to handle manuscripts in a wide range of topics.

And that does it for the Executive Director's Report. MLA is an exceptional organization, and it is a true pleasure to serve you as our Executive Director.

Kris Alpi: Thank you, Kevin.

### Annual Report

Kris Alpi: The next order of business is to present the 2021-2022 Annual Report. The MLA annual reports are valuable for all of us to read. You may discover parts of MLA you did not know, or incredible things your colleagues have enabled. Take time to read them, even if it is just the executive summaries. Those reports show the immense diversity of our communities and programs. They are also part of MLA's archives, so if your name is in there, you are officially recorded for posterity. This slide shows all of the MLA components that contribute to this comprehensive document. The reports are available on MLANET, along with reports from past years.

### Recognition of Retiring Directors

Kris Alpi: Those who are nominated each year as potential members of the MLA Board of Directors are selected by virtue of their experience and reputation to serve the association. But few can imagine beforehand the level of commitment that election to the board requires. And it seemed like even more so this year…

Directors who have completed their term on the MLA Board have served our association with enthusiasm, dedication, grace, good humor, and perseverance! The association and the Board of Directors express our appreciation and recognize each of you today for the extraordinary work and thoughtful leadership you have provided during your term of service. Thank you for a job well done.

- Donna R. Berryman, AHIP
- Sally Gore
- Meredith I. Solomon, AHIP

### Recognition of Immediate Past President

Kris Alpi: I would like to express my sincere gratitude to

Lisa Traditi, AHIP, MLA's 2020-2021 president.

Dearest Lisa,

It's been three very long years, and I was lucky to be learning from you for the past two of them. All of us enjoyed being able to celebrate with you in person in New Orleans and meet Frank who can finally see an end to starting your Friday mornings with MLA Executive Committee meetings. Hooray! We were also happy to give you a crystal gavel to commemorate your presidency in New Orleans.

Throughout your presidency you were a voice of calm, reason, inspiration and action, helping the association and our members maneuver through the troubled times of the pandemic and incidents of social injustice. Your leadership resulted in the release of several MLA, caucus, and chapter statements responding to the death of George Floyd and reaffirming MLA's commitment to diversity, equity, and inclusion.

You led the Development and release of MLA's Statement of Appropriate Conduct which strengthens our commitment to provide an environment of mutual human respect in which diverse participants may learn, network, and enjoy their interactions with colleagues.

You successfully oversaw the creation of the 2021 conference experience that enhanced the attendee experience. The conference included a virtual component that built upon the success of the MLA 2020 the MLA 2020 vConference and Exhibits, with improvements based on member feedback and lessons learned.

When the University of Connecticut Health Center (UCHC) announced it would cease operations of its Electronic Funds Transfer System (EFTS), you and the Board of Directors supported MLA taking over operations. Enhancements were made to the platform, and this hugely successful effort has provided seamless support to hundreds of libraries that would have had no viable alternative to cost-effectively exchange funds for publications accessed through inter-library loan.

Lisa, thank you for all you do for MLA and the profession. You are an inspiration for all of us.

Lisa Traditi: Kris, thank you for your kind words. It was an honor and privilege to serve the members of MLA. Thank you.

Kris Alpi: Thank you, Lisa and please continue to enjoy your retirement!

### Announcement of Election Results

Kris Alpi: The MLA 2022 election was conducted from March 14 to April 6, 2022. Voting statistics can be seen on your screen. Election results were announced on April 13, 2022, in MLAConnect.

Following are the election results. Nine individuals were elected for a one-year term to the Nominating Committee. Their names appear on your screen:

- Joy Summers-Ables, AHIP
- Lisa Huang
- Elizabeth Laera, AHIP
- Carolyn Ching Dennison, AHIP
- Aidy Weeks
- Ryan Harris, AHIP
- Susan Lessick, AHIP, FMLA
- Francisco J. Fajardo, AHIP
- Sylvia McAphee

Amy Blevins, AHIP, was elected to serve as President-Elect. Welcome back to the Board, Amy. Amy was MLA's Treasurer from 2017-2019.

Congratulations also to Tony Nguyen [GWEN], AHIP and Emily Hurst, AHIP who were each elected by the membership for a three-year term to the MLA Board of Directors. PJ Grier was elected by Chapter Council to serve as its 2022 to 2025 Chair and in this role he also will serve a three-year term on the MLA Board of Directors. Welcome Tony, Emily, and PJ.

Kris Alpi: Now, it's time for my year as MLA president to come to a close.

It is my honor and pleasure to introduce your 2022-2023 President, Shannon Jones, AHIP, FMLA, Director of Libraries at the Medical University of South Carolina, and Director of Region 2 of the National Network of Libraries of Medicine.

Shannon began her career in health sciences as a Fellow with the National Library of Medicine in September 2002. Since then, she has worked exclusively in health science libraries beginning in 2004 as an education and outreach librarian at Tompkins-McCaw Library for the Health Sciences at Virginia Commonwealth University under the leadership of former MLA President Teresa Knott, AHIP, FMLA.

Shannon has held multiple leadership positions within MLA, including founding the New Members Special Interest Group and serving as a Co-Convener, African American Medical Librarians Alliance (AAMLA).

Shannon's reputation as a leader in the profession is widely recognized, especially in the areas of mentoring, and in diversity, equity, and inclusion. She has coordinated AAMLA's Virtual Book Discussion Group which has grown from 50 participants to 200 in the last two years. She is a frequent speaker and panelist at conferences to discuss topics on leadership, career growth, and diversity, equity, and inclusion. Please watch for the July issue of JMLA to learn more about Shannon.

And now I am thrilled to pass the gavel to my distinguished colleague Shannon Jones, AHIP, FMLA.

[Kris puts down her gavel; Shannon takes up her gavel (right hand moving in towards screen)]

### Recognition of Outgoing President

Shannon Jones: Kris, I am pleased to thank you on behalf of the MLA membership for your leadership during this challenging Presidential year. We were impressed to hear about all the activities you undertook during your year as President, and I encourage everyone to listen to the opening session recording of Kris' Presidential Address if you missed the chance to hear it live. To highlight a few, you have been a strong advocate for the “voice of the member” striving to ensure that everyone's voice is heard. Your diligence and commitment have led to the establishment of the hospital library caucus advocacy initiative to develop resources for hospital and health care administrators highlighting the mission-critical roles that health sciences information professionals provide in healthcare settings. One key outcome is the statement calling on hospital and health system executives to fund libraries and library staff. Another initiative seeks to ensure that librarians and information professionals earn authorship on evidence synthesis publications, such as guidelines and systematic reviews, for their intellectual contribution to published works. Kris, you have distinguished yourself in the area of research, generously sharing your knowledge and skills, most recently supporting the assessment of MLA's new communities' governance model. Your work with the MLA InSight Initiative has helped lay the groundwork for the Development of MLA's first-time Collection Development Symposium that was held in New Orleans last week. Your energy, drive, and commitment to MLA and our members is reflected in everything you do. We were happy to give you a silver cup, our token of appreciation for all you have led us in accomplishing this year, last week in New Orleans. Kris, raise your cup to the camera for all to see.

I hope you will display this cup proudly because it symbolizes a year when we in MLA broadened our opportunities to build our future under your leadership.

Kris Alpi: Thank you, Shannon. And thank you to all of our MLA community who made this year possible.

### Presentation of the 2022-2023 Board of Directors

Shannon Jones: MLA members, I am very pleased to present your 2022-2023 Board of Directors.

Congratulations to all of the Directors on being elected! If our names are not yet familiar to you, especially those of you who may have joined MLA after the election, I hope that you will look us up and connect with us via MLANET. And here are our photos to help put faces with our names as you see us either at future virtual forums, committee, caucus, chapter or domain hub meetings, or in-person next year at MLA.

I look forward to working together and thank you to the Board members for your commitment to MLA and dedication to the profession.

### Resolutions

Shannon Jones: I now have the honor to finish the remaining items of business before we adjourn. Next are resolutions.

We have no resolutions at this time and will move onto new business.

### New Business

Shannon Jones: Does anyone have new business to bring before the assembly?

Please raise your hand if you have new business. You have 20 seconds to raise your hand.

[No raised hands]

### Adjourn

Shannon Jones: Let's welcome back Heather Holmes, the MLA Board Secretary for our final item of business. Hi, Heather.

Heather Holmes: Before I call for the meeting to adjourn, I'd like to say how happy I am for our next order of business. Most of you know that our incoming President Shannon Jones is my boss, and I could not be any prouder to get to work alongside her every day. Not a day goes by that I don't learn something from her, and I know this coming year is going to be great for MLA under her leadership.

And now, for our final item of business, I move to adjourn the 2022 MLA Annual Business Meeting.

Shannon Jones: OK, it has been moved to adjourn, but please stick around afterwards for my inaugural Presidential Address.

I propose to approve the motion by unanimous consent. Any member may object, in which case we will call for discussion and then vote. Please raise your hand if you object. You have 20 seconds to do so. Not seeing any objections, we will not proceed to a vote.

The motion is passed, and the meeting is adjourned. Thank you in advance for staying with us for this next piece of our program.

## MLA 2021/2022 INCOMING PRESIDENTIAL ADDRESS (PLENARY SESSION)

Shannon Jones: Good afternoon colleagues! I am eternally grateful for each of you who showed up today to hear my inaugural address. It was so good to see so many of you in New Orleans last week, and I am equally excited to welcome those who attended the virtual conference.

When I attended my first MLA meeting in May 2003, I never imagined that I would be embarking on this journey of serving as MLA's second Black President.

Arriving at this point in a career is impossible without the investment of time and energy of a dedicated village. An African proverb says it takes a village to raise a child. I believe that the same is true for grooming an individual for leadership at the highest level of the Association.

I will begin my comments today by thanking my village. I offer sincere thanks to the information professionals who believed in me, supported my goals, fed my dreams, pushed me towards greatness, corrected me when I was wrong, laughed with me, spoke life into me, nominated me, sponsored me, and advocated for me when I was not in the room. I am grateful they saw, heard, affirmed, and, most importantly, included me in opportunities to help advance my career.

First is the staff of the Medical University of South Carolina Libraries. Leading at this level is not possible without the support of a strong library team. The faces you see on the screen are the dedicated information professionals who comprise the library I lead. I could not do this work without their support and commitment to keeping the ship afloat.

Second, are the mentors who modeled the way. A quote from John C. Maxwell says A leader knows the way, goes the way, and shows the way. A sincere thanks to Tom Basler, Sandra Franklin, Teresa Knott, Sandra Martin, Beverly Murphy, Jean Ship.m.an, MJ Tooey, and John E. Ulmschneider. I would not be where I am today if it were not for the leaders who pushed, encouraged, loved, supported, and challenged me to be the best librarian possible.

Third, I was encouraged to find my community within MLA when I was new to the Association. For me, that community has been the African American Medical Librarians Alliance Caucus (AAMLA). Since 2002, I have worked with fellow AAMLA members to solidify the legacy of Black librarians in MLA's history. I am grateful for their presence in this space and their support of me, and most importantly, their commitment to operationalizing AAMLA's mission of providing leadership and support on issues such as recruiting and retaining diverse librarians, developing mentoring and leadership for minority librarians, and creating a repository of information on the expert skills and special abilities of minority librarians in the Medical Library Association (MLA), particularly those of African American descent.

Fourth, I have served on the MLA Board of Directors since 2018 and have had the opportunity to watch and learn from four AMAZING leaders. Anyone who has held a volunteer position knows that Service Requires Sacrifice. A quote from Jon Gordon says, “When you love, you serve, and when you serve, you sacrifice. Service requires a sacrifice of something. Whether it's time, energy, money, love, effort, or focus, serving others always costs you something, but with service and sacrifice, you gain so much more.” So I thank Beverly Murphy, Julia Esparza, Lisa Traditi, and Kris Alpi for their service to our Association. I have gained so much from watching you all lead and give of yourselves for the greater good of MLA. So I thank you too.

Fifth, I humbly stand on the shoulders of giants in Black librarianship. Their sacrifice, advocacy, resilience, and power made it possible for me to stand before you as the second Black MLA President, and I do not take it lightly. I had the opportunity to stand in the presence of Dr. EJ Josey, who was from my hometown in Norfolk, VA. I stand in reverence to the Black library luminaries who came before me. Thank you to Dr. EJ Josey, Dr. Jessie Carney Smith, Dr. Virginia Lacy Jones, Joseph H. Reason, Clara Stanton Jones, Dr. Eliza Atkins Gleason, and MLA's own Dr. Gwendolyn Cruzat. I am eternally grateful for the path they blazed for countless Black librarians like myself to find our place in the profession.

Last and certainly not least, everyone who showed up today. I am grateful for your continued commitment to MLA, and I look forward to getting to know you this coming year.

I would like to share a little about myself and my journey to the MLA presidency.

Since 2002, MLA has been my professional home. As a new librarian, I was eager to be a part of an organization where I could share my time and talent for the greater good of health sciences librarianship. This quote from Martin Luther King, Jr. states, “Everybody can be great…because anybody can serve. You don't have to have a college degree to serve. You don't have to make your subject and verb agree to serve. You only need a heart full of grace. A soul generated by love.” I approached my MLA volunteerism with greatness in mind. I sought to be an excellent librarian, colleague, friend, mentee, mentor, collaborator, and an overall great person. My MLA volunteerism has allowed me to do great things while in service to the profession and others. As I reflect over almost twenty years of service to MLA, I am grateful for opportunities that have positioned me to:

1. Engage in work that impacts the Association's future and sustainability.
2. Find my professional voice and positionality in health sciences librarianship.
3. Enhance collaboration, teamwork, leadership, and advocacy skills.
4. Cultivate meaningful relationships with a diverse group of information professionals.
5. Support legacy-building efforts of BIPOC [Black, Indigenous, People of Color] librarians in the Association and health sciences librarianship.
6. Level up my ability to interact with integrity, respect, authenticity, empathy, compassion, and joy.

I am also a Girl Scout and a troop leader. For over 20 plus years, I have lived by the Girl Scout Promise and Law, which characterizes a leader as someone who is:

- friendly and helpful,
- considerate and caring,
- courageous and strong,
- responsible for what they say and do,
- respect themselves and others,
- Respect authority
- uses resources wisely,
- and makes the world a better place.

It is these characteristics that I work diligently to showcase in my daily interactions as a leader at MUSC, a member/volunteer of the MLA, and within my community as a Troop leader.

One of the biggest lessons I have learned from being a girl scout is to “leave a place better than you found it.” I operationalize this concept by working to make the lives of the people with whom I interact better because I was in their life.

I am a dog and plant mom - Meet my fur babies who rescued me: The oldest of the two is Cooper, who is six years old. We call him “Coop Loop,” and then there is one-year-old Ramsey affectionately known as “Ram Ram” or “Rambunctious.”

Thanks to the wisdom of Aidy Weeks, Beverly Murphy, and Kelsa Bartley, I developed a love for plants during the pandemic. Through their encouragement, I now have a growing family of houseplants that I have nurtured for almost two years after years of being a card-carrying member of the black thumb group. Through trial and error, my green thumb has flourished, and my plant babies are thriving.

Now that I've told you who I am, let's shift to looking at the mindset that will guide my efforts in the coming year.

As I contemplated what I would say to you all today, my thoughts went back to March 2020. For me, two pivotal incidents happened in March 2020: The murder of Breonna Taylor and when South Carolina Governor Henry McMaster told all state workers to go home and work remotely. Then my thoughts took me to George Floyd's murder in May 2020. All of this happened with the COVID-19 pandemic raging in the background.

Then I reviewed the statement I wrote in response to the prompts the Nominating Committee gave to candidates for MLA President.

Almost three years into the pandemic, we can all admit that life during COVID-19 has caused trauma for many of us, personal or professional. From 2020 to 2022, we experienced a tense presidential election, isolation, social distancing, masking, shutdowns, budget reductions, furloughs, and layoffs. We pivoted unexpectedly to remote work and launched new services; some of you fulfilled the role of teacher and caregiver, fought fake news and misinformation, and served the Association, despite the disruption that COVID-19 ushered into our lives.

I heard weariness in my colleagues' voices and saw fatigue in their eyes and posture. While it saddened me, it also inspired me to bring out my best self as I watched colleagues put on brave faces and not acquiesce to any anxieties they may have felt.

It made me think of all the traumas we experienced this year. We do not know people's stories, what they deal with outside of work, or what they are shouldering.

1. This reflection led me to the concept of radical empathy as “The New Normal.” Terri Givens, author of *Radical Empathy: Finding a Path to Bridging*, says radical empathy means having two kinds of empathy: emotional empathy, or feeling how another person feels, and cognitive empathy, or understanding how another person sees the world. She said it also involves taking action to change structural inequality beyond just being nice to other people. In the book, she offers six steps we can take individually to move towards radical empathy. She says we must have:
  - A willingness to be vulnerable.
  - Becoming grounded in who you are.
  - Opening yourself to the experiences of others.
  - Practicing empathy.
  - Taking action.
  - Creating change and building trust.
2. Diversity, Equity, and Inclusion
  - MLA is not the same Association it was when I attended my first conference in May 2003. At that conference, I was struck by the lack of diversity amongst the members.
  - MLA is a much-improved Association from when I joined in 2002. I am proud of our DEI efforts, as it's been a long time coming. We are not where we need to be, but we are actively working on getting it right for ALL of our members. We are doing the work to walk the walk, and I am proud of us.
3. Building a Better Future
  - In 2023, MLA will celebrate its 125th anniversary, which I look forward to celebrating with you in Detroit, MI. I am most excited about the Association's stated commitment to ensuring that the voices of all MLA members are represented within the historical narrative. I look forward to contributing to the work MLA members do to capture the voices, perspectives, contributions, and history of members traditionally left on the margins of the Association's life.
4. Wellness and Wellbeing
  - That said, the reality is that we cannot pour from empty vessels, nor can we do more with less realistically. Disconnecting to replenish our minds and bodies is essential for improving our professional practice. Whether it's getting lost in the pages of a book, taking a walk, listening to music, coloring, cooking, painting, or knitting, MLA members should adopt a self-care strategy that suits their interests and lifestyle.

Now, if we are to accelerate any of this work, I need you to do as Pop Icon Beyonce says and get “In Formation.” I need you to walk in lockstep, hand in hand with me, to do this work.

I commit to you as Your president to work with you on the frontlines to advocate for the greater good of all MLA members. It will take the collective WE to move the needle with the ambitious goals that MLA has set. In the words of my colleague, past president Beverly Murphy: I am MLA, You are MLA, WE are MLA.

I thank you for the opportunity to serve as your president. I know that the future isn't always clear; however, I know that together, we will do everything we can to make the future of MLA fantastic. Thank you, everyone.

I invite you to join me on this journey!

## OTHER PLENARY SESSIONS

(The following plenaries were also livestreamed for virtual attendees)

### Monday, May 5, 2022, John P. McGovern Award Lecture.

Keynote Speaker: Mona Hanna-Attisha, MD, MPH, FAAP

The 2022 McGovern Lecture will be a structured conversation with our speaker and the 2022 National Program Committee Cochairs.

Mona Hanna-Attisha, MD, MPH, FAAP, founder and director, Michigan State University and Hurley Children's Hospital Pediatric Public Health Initiative, Flint, Michigan. Dr. Hanna-Attisha was named one of Time magazine's 100 Most Influential People in the World and recognized as one of USA Today's Women of the Century for her role in uncovering the Flint water crisis and leading recovery efforts. She is the author of the widely acclaimed and bestselling book What the Eyes Don't See: A Story of Crisis, Resistance, and Hope in an American City.

### Wednesday, May 4, 2022, Janet Doe Lecture: Health Science Libraries in the Emerging Digital Information Era: Charting the Course

Keynote Speaker: Michael R. Kronenfeld, AHIP, FMLA

Michael Reed Kronenfeld, MLS, MBA, AHIP, FMLA is University Librarian Emeritus, A.T. Still University of the Health Sciences. In March 2021, his book titled A History of Medical Libraries and Medical Librarianship: From John Shaw Billings to the Digital Era was published by Rowman and Littlefield as part of MLA's book publishing program. This Lecture is primarily based on this research.

### Symposium on Collection Development and Resource Sharing

Symposia sessions were held in person only throughout the conference.

*Wednesday, May 4, 2022*

- Negotiation Strategies to Empower You in Libraries and Life: A Hands-On Workshop
- Riding the Waves Together: A Librarian and Publisher Conversation

*Thursday, May 5, 2022*

- DEI&A Initiatives & Challenges: Librarian and Publisher Lessons from the Frontlines
- Staying Ahead of the Future: Developing Your Library's Collection Philosophy and Policy

*Friday, May 6, 2022*

- Think Like a Lawyer: A Socratic Seminar on Copyright Law
- From Selecting to Weeding: Getting “Hands-On” with Tools that Help Resources Become a Collection

## PROGRAM SESSIONS

Program sessions were available in an on-demand viewing format, and a live format.

On-demand viewing for 47 papers, and 29 lightning talks began on April 27, 2022.

Sponsors: Springer Nature, Clinical Key, and the National Library of Medicine presented 4 On-demand sessions.

During the face-to-face portion of the annual meeting, there were 16 immersion sessions, 59 papers, and 20 lightning talks. The live immersions sessions included interactive breakout sessions, Q & A, and virtual chat with presenters.

Paper abstracts that were scheduled to be presented are available on the MLA '22 website. The final version of the abstracts reflecting only those presented at the meeting is included as an online-only supplemental file to the January 2023 issue of the Journal of the Medical Library Association.

## POSTER SESSIONS

The Poster Gallery featured 49 posters in an on-demand viewing format beginning April 27, 2022. These included audio presentations and virtual and/or chat Q & A sessions with the authors. There were 50 posters that were presented live in New Orleans, LA over two sessions: Thursday, May 5, 2022, 12:30-1:30 p.m. and Friday, May 6, 2022, 12:30-1:30 p.m.

Poster abstracts that were scheduled to be presented are available on the MLA '22 meeting website. The final version of the abstracts reflecting only those posters presented at the meeting is included as an online-only supplemental file to the January 2023 issue of the Journal of the Medical Library Association. The actual posters are available online in the MLA '22 meeting website.

### MLA Research Training Institute (RTI) Poster Ignite Session

The MLA Research Training Institute had a dedicated in person poster session.

*Thursday, May 5, 2022*

- The Paradox of Disinformation in Hospital Libraries, Jess Callaway, AHIP
- Knowledge of the Systematic Review Process Among Health Sciences Faculty, Amy Corder
- Examining the Institutional Review Board Process: A Content Analysis of IRB Submission and Approval Documents of RTI Participants, Andrea Dater
- Exploring DNP Student Information Literacy Competence for Evidence-Based Practice, Jennifer DeBerg
- What Do Systematic Searchers Need from Search Interfaces?, Andy Hickner
- Technical Services Librarians and MLA: Attitudes and Perceptions, Toni Hoberecht
- Stigmatized Mental Health Content on TikTok: A Qualitative Content Analysis, Valerie Lookingbill
- Is Our Hospital Library Website Efficiently Meeting the Needs of Our Clinicians for Their Effective Practice of EBM/EBP?, Michele Mason-Coles
- Osteopathic Medical Students Attitudes Towards Research: A Mixed-Methods Study, Molly Montgomery
- Examining the Role of the Health Science Librarian in Supporting LGBTQ+ Information Literacy Among Health Science Students, Residents, Fellows, or Faculty, Laura Murray
- Tenure, Promotion, Appointment, Oh My! The Processes of Academic Health Sciences Libraries, Erin Reardon
- The Scholarly Activity of GME Trainees: Early Stages of a Research Project That Analyses Publication Patterns Julia Stumpff

## OTHER MEETINGS AND EVENTS

The following MLA Chapter meetings were held prior to, during, and after MLA '22: Hawaii-Pacific Chapter Meeting, April 22, 2022; South Central Chapter Meeting, September 22-27, 2022; Southern Chapter Meeting, October 19-23, 2022; Pacific Northwest Chapter Meeting, October 20-21, 2022; Midwest Chapter Meeting, October 22-24, 2022; Mid-Atlantic Chapter Meeting, October 23-25, 2022; Upstate NY and Ontario Chapter Meeting, October 26-28, 2022; Midcontinental Chapter Meeting, October 27-28, 2022; Liberty Chapter Meeting, Nov. 1, 2022; North Atlantic Health Sciences Libraries, Inc. Conference, November 6-8, 2022.

The following MLA Caucus meetings were held virtually prior to and just after MLA ‘22: African American Medical Library Alliance Caucus Business Meeting, April 25, 2022; Animal and Veterinary Information Specialist Caucus Information Meeting, April 19, 2022; Basic Science Caucus Business Meeting, April 14, 2022; Cancer Librarians Caucus Business Meeting, April 28, 2022; Clinical Librarians and Evidence Based Practice Caucus Meeting, February 17, 2022; Collection Development Caucus Business Meeting, April 28, 2022; Complementary and Integrative Health Caucus Business Meeting, May 26, 2022; Consumer and Patient Health Information Services Caucus Business Meeting, July 18, 2022; Data Caucus Business Meeting, April 18, 2022; Dental Caucus Workshop, May 25, 2022; Federal Libraries Caucus Business Meeting, April 20, 2022; Gaming in Adult Learning Caucus Business Meeting, April 25, 2022 (joint with Technology in Education Caucus); Health Association and Corporate Librarians Caucus Business Meeting, April 25, 2022; Health Humanities Caucus Business Meeting, May 12, 2022; History of the Health Sciences Caucus Business Meeting, April 11, 2022; Hospital Library Caucus Business Meeting, April 21, 2022; International Cooperation Business Meeting, June 21, 2022; Interprofessional Education and Practice Caucus Business Meeting, April 27, 2022; Latinx Caucus Business Meeting, April 22, 2022; Leadership &amp; Management Caucus Business Meeting, June 10, 2022; LGBTQIA+ Caucus Business Meeting, April 18, 2022; Libraries in Health Sciences Curriculums Caucus Meeting, February 10, 2022; Medical Informatics Caucus Business Meeting, April 4, 2022; Medical Library Education Caucus Business Meeting, April 28, 2022; New Members Caucus Business Meeting, April 7, 2022; Nursing &amp; Allied Health Resources and Services Caucus, April 22, 2022; Osteopathic Libraries Caucus Business Meeting, April 29, 2022; Pediatric Librarians Caucus Business Meeting, April 26, 2022; Public Health/Health Administration Caucus Business Meeting, April 8, 2022; Public Services Caucus Business Meeting, April 26, 2022; Resource Sharing Caucus Business Meeting, April 28, 2022; Scholarly Communications Caucus Business Meeting, April 28, 2022; Systematic Reviews Caucus Business Meeting, June 15, 2022; Technical Services Caucus Business Meeting, May 10, 2022; Technology in Education Caucus Business Meeting, April 25, 2022 (joint with Gaming in Adult Learning Caucus); UX Caucus Business Meeting, April 7, 2022; Vision Science Caucus Business Meeting, April 11, 2022.

## OPEN FORUM

Thursday, May 5, 2022

Vision 2048 Task Force Open Forum: Envision the Future of Health Sciences Libraries

## NATIONAL LIBRARY OF MEDICINE UPDATE

The National Library of Medicine (NLM) Update and Q&A took place on Friday, May 6, 2022, from 9:00-10:20AM and was also livestreamed for virtual attendees.

## OTHER SPECIAL EVENTS AND RECEPTIONS

Wednesday, May 4, 2022, 7:30-8:55 a.m.

- New Members/First Time Attendee Program & Networking

Wednesday, May 4, 2022, 12:00-1:30 p.m.

- MLA Fellows Meeting and Networking Session

Wednesday, May 4, 2022, 12:30-1:45 p.m.

- A Gathering of Caucuses and Domain Hubs: Celebrating Our Communities Lunch

Wednesday, May 4, 2022, 4:30-5:15 p.m.

- MLA Leaders' Recognition and International Visitor Reception (by invitation)

Thursday, May 5, 2022, 10:30-11:50 a.m.

- Public Health Focus Panel (with Dr. Mona Hanna-Attisha)

Thursday, May 5, 2022, 2:00-3:20 p.m.

- Vision 2048 Working Session: Envision the Future of Health Sciences Libraries

Thursday, May 5, 2022, 3:30-4:50 p.m.

- Living Library

Thursday, May 5, 2022, 6:30-8:30 p.m.

- Networking and Gaming Night

Friday, May 6, 2022, 3:30-3:50 p.m.

- Closing Session

## EXHIBIT HALL

The Exhibit Hall was home to 45 vendors who presented various demonstrations of their products. The Exhibit Hall began with an opening reception on Wednesday, May 4, 2022, 5:30-7:30P.M. Central time that was sponsored by NEJM Group. The Exhibit Hall was open on Thursday, May 5, 2022, 9:00AM-5:00P.M. and Friday, May 6, 2022, 9:00AM-2:00P.M.

Exhibitors held both sunrise seminars, lunch and learns, as well as technology showcases to highlight new products.

### Thursday, May 5, 2022

Sunrise Seminars

- Wolters Kluwer
- Covidence – The Future of Evidence: What Have We Learnt Through the Pandemic
- Elsevier

Lunch and Learns

- Clarivate
- EBSCO – The Latest from EBSCO (by invitation only)
- Elsevier

Technology Showcase

- Rittenhouse – The R2 Digital Library from Rittenhouse: Evidence-Based Collection Development for Health Sciences
- Clarivate – What's new with EndNote
- Covidence – Systematic Review Technology Now and for the Future
- BMJ – Wish you could improve research quality and increase output?

### Friday, May 6, 2022

Sunrise Seminars

- Springer Nature (by invitation only)

Lunch and Learns

- Wolters Kluwer – Translate Evidence into Practice to Improve Patient Care (by invitation only)
- Elsevier-Clinical Key – Harnessing the Headwinds of Change (by invitation only)

Technology Showcase

- Clarivate – Getting the Most from Your Research
- Elsevier – Exciting Developments with Digital Commons and Digital Commons Data

## CONTINUING EDUCATION COURSES

Tuesday, May 3, 2022

CE100 Essential Searching Skills for Librarians on Systematic Review Teams

CE400 Do You Want to Be a Library Director?

## RESOURCES AND SERVICES

The online itinerary planner sponsored by Wolters Kluwer allowed attendees to peruse programs and events online. Live streaming was available on Twitter using the hashtag #mlanet22. The annual meeting blog posts are available on the MLA website. The MLA Professional Recruitment and Retention Committee (PRRC) is pleased to sponsor the MLA '22 Virtual Resume Clinic.

